# HMGB1 Promotes the Development of Pulmonary Arterial Hypertension in Rats

**DOI:** 10.1371/journal.pone.0102482

**Published:** 2014-07-17

**Authors:** Yukari Sadamura-Takenaka, Takashi Ito, Satoshi Noma, Yoko Oyama, Shingo Yamada, Ko-ichi Kawahara, Hiromasa Inoue, Ikuro Maruyama

**Affiliations:** 1 Department of Pulmonary Medicine, Kagoshima University Graduate School of Medical and Dental Sciences, Kagoshima, Japan; 2 Department of Systems Biology in Thromboregulation, Kagoshima University Graduate School of Medical and Dental Sciences, Kagoshima, Japan; 3 Department of Laboratory and Vascular Medicine, Kagoshima University Graduate School of Medical and Dental Sciences, Kagoshima, Japan; 4 Shino-Test Corporation, Sagamihara, Japan; 5 Department of Biomedical Engineering, Osaka Institute of Technology, Osaka, Japan; Vanderbilt University Medical Center, United States of America

## Abstract

**Rationale:**

Pulmonary arterial hypertension (PAH) is characterized by increased pulmonary vascular resistance leading to right ventricular failure and death. Recent studies have suggested that chronic inflammatory processes are involved in the pathogenesis of PAH. However, the molecular and cellular mechanisms driving inflammation have not been fully elucidated.

**Objectives:**

To elucidate the roles of high mobility group box 1 protein (HMGB1), a ubiquitous DNA-binding protein with extracellular pro-inflammatory activity, in a rat model of PAH.

**Methods:**

Male Sprague-Dawley rats were administered monocrotaline (MCT). Concentrations of HMGB1 in bronchoalveolar lavage fluid (BALF) and serum, and localization of HMGB1 in the lung were examined over time. The protective effects of anti-HMGB1 neutralizing antibody against MCT-induced PAH were tested.

**Results:**

HMGB1 levels in BALF were elevated 1 week after MCT injection, and this elevation preceded increases of other pro-inflammatory cytokines, such as TNF-α, and the development of PAH. In contrast, serum HMGB1 levels were elevated 4 weeks after MCT injection, at which time the rats began to die. Immunohistochemical analyses indicated that HMGB1 was translocated to the extranuclear space in periarterial infiltrating cells, alveolar macrophages, and bronchial epithelial cells of MCT-injected rats. Anti-HMGB1 neutralizing antibody protected rats against MCT-induced lung inflammation, thickening of the pulmonary artery wall, and elevation of right ventricular systolic pressure, and significantly improved the survival of the MCT-induced PAH rats.

**Conclusions:**

Our results identify extracellular HMGB1 as a promoting factor for MCT-induced PAH. The blockade of HMGB1 activity improved survival of MCT-induced PAH rats, and thus might be a promising therapy for the treatment of PAH.

## Introduction

Pulmonary arterial hypertension (PAH), characterized by increased pulmonary vascular resistance leading to right ventricular failure, is a progressive disease with a poor prognosis [1]. The main pathological finding related to PAH is an abnormal proliferation of endothelial cells and smooth muscle cells resulting in obstruction of small pulmonary arteries [2], [3]. Recent studies have suggested that inflammatory processes are involved in the initiation and progression of PAH and the remodeling of the pulmonary vasculature [4], [5]. Indeed, PAH is often associated with inflammatory diseases, such as connective tissue diseases and human immunodeficiency virus (HIV) infection [6], [7]. Pro-inflammatory cytokines and chemokines, such as tumor necrosis factor-α (TNF-α), interleukin-8 (IL-8), monocyte chemotactic protein-1 (MCP-1), and regulated upon activation, normal T-cell expressed, and secreted (RANTES), contribute to the recruitment of leukocytes, the induction of endothelin-1, and the proliferation of smooth muscle cells [7]–[10]. Thus, although it is widely known that inflammation plays a prominent role in the development of PAH, molecular and cellular mechanisms driving inflammation remain poorly defined.

High mobility group box 1 protein (HMGB1), a ubiquitous DNA-binding protein, triggers inflammatory responses when released into the extracellular space. In response to stress, HMGB1 is actively secreted from immunocompetent cells and passively released from damaged cells [11]. Extracellular HMGB1 transduces cellular signals by acting on pattern recognition receptors, such as receptor for advanced glycation end products (RAGE) and toll-like receptors, expressed on target cells, inducing the release of pro-inflammatory cytokines [12], the maturation of dendritic cells [13], and the chemotaxis and proliferation of smooth muscle cells [14]. Extracellular HMGB1 has been reported to contribute to the pathogenesis of acute and chronic inflammatory diseases, including sepsis [15], rheumatoid arthritis [16], atherosclerosis [17], thrombotic disorders [18], [19], kidney impairment [20], and acute lung injury [21]. Here, we show that HMGB1 is released into the extracellular space during the early stage of monocrotaline (MCT)-induced PAH and contributes to the development of PAH in rats. Our findings provide novel insight into the molecular basis of PAH and a potential therapeutic target.

## Materials and Methods

### Animals

Experiments involving animals were approved by the Institutional Animal Care and Use Committee of Kagoshima University, Kagoshima, Japan, and were performed in accordance with the guidelines of the Frontier Science Research Center, Kagoshima University, Kagoshima, Japan. Male Sprague-Dawley (SD) rats (240–300 g in body weight) were housed under a 12∶12 hour light-dark cycle in a specific pathogen**-**free environment and had free access to food and water. Rats were randomly divided into 4 groups, and were given a single intraperitoneal (i.p.) injection of 0.9% saline or 60 mg/kg of MCT. MCT-injected rats were treated for 2 weeks by i.p. injection of either vehicle, anti*-*HMGB1 neutralizing IgY (2 mg/kg) (Shino-test, Sagamihara, Japan), or control IgY twice a day. For survival studies, rats were monitored twice daily for signs of disease, and were considered lethally induced on the day they were no longer able to take food or water or they lost more than 20% of their body weight, at which time they were humanely euthanized by exsanguination under anesthesia. Survival was monitored for up to 6 weeks after MCT injection.

### Hemodynamic measurement

Hemodynamic measurements were carried out as described previously [22]. Briefly, a polyethylene catheter was inserted into the right ventricle (RV) for hemodynamic measurements after the animals were anesthetized with ketamine (60 mg/kg, i.p.) and sodium pentobarbital (20 mg/kg, i.p.). RV systolic pressure (RVSP) was measured with a polygraph system (AP-601G, Nihon Kohden Co., Tokyo, Japan). The ratio of RV weight to body weight (RV/BW) and the ratio of RV weight to left ventricular (LV) plus septal weight (RV/LV+S) were calculated as indices of RV hypertrophy, as described previously [23]. For the experiments with neutralizing antibody, hemodynamic studies were performed 21 days after MCT injection. After RVSP was measured, blood was collected from caudal vena cava of the rats.

### Morphometric analysis of pulmonary arteries

Isolated left lungs were inflated with 10% formalin, fixed, and then embedded in paraffin. Hematoxylin and eosin (H&E) staining and Elastica van Gieson staining were performed for morphometric analysis. The external diameter and medial wall thickness of the pulmonary arteries were measured in 20 muscular arteries (ranging in size from 25–100 µm in external diameter) on Elastica van Gieson–stained sections. For each artery, medial wall thickness was expressed as follows: % wall thickness = [(medial thickness×2)/external diameter]×100.

### Immunohistochemical analysis

Immunohistochemical analysis was carried out as described previously [20]. Briefly, paraffin sections were deparaffinized and endogenous peroxidase activity was blocked by treatment with 0.3% H_2_O_2_ solution for 15 min. The sections were then blocked with 2% goat serum for 30 min. The sections were incubated with 2 µg/mL of anti-HMGB1 rabbit polyclonal IgG (Shino-Test Corporation, Kanagawa, Japan) or anti-smooth muscle actin mouse monoclonal IgG (Sigma-Aldrich, St Louis, MO) overnight at 4°C. The sections were washed with tris-buffered saline containing 0.2% Tween-X100 (TBST), and then were incubated with Histofine simple stain MAX-PO for 60 min at room temperature (RT). The sections were washed and further incubated with the 3-amino-9-ethylcarbazole substrate-chromogen system (Nichirei, Tokyo, Japan) for 10 min at RT. As a negative control, each isotype antibody from the same species was used instead of the primary antibody. The sections were counterstained with Mayer’s hematoxylin, mounted in Aquatex (Merck KGaA, Darmstadt, Germany) and examined with a BH2 light microscope (Olympus, Tokyo, Japan). For immunofluorescent analysis, the sections were probed with anti-HMGB1 antibody followed by Alexa-Fluor 488-conjugated goat anti-rabbit IgG (Invitrogen, Carlsbad, CA). Nuclei were stained with 4′,6-diamidino-2-phenylindole (DAPI), and the sections were analyzed using an LSM700 confocal laser microscope (Zeiss, Oberkochen, Germany).

### Measurement of HMGB1, MCP-1, TNF-α, IL-1β, and endothelin-1 in serum and BALF

HMGB1 (Shino-Test Corporation), MCP-1 (BioSource, Camarillo, CA, USA), TNF-α (BioSource), IL-1β (Invitrogen), and endothelin-1 (Bachem, Bubendorf, Switzerland) levels in rat BALF and serum were measured using ELISA kits in accordance with the manufacturer’s instructions.

### Statistical analysis

All data are expressed as mean ± SEM. Comparisons of parameters among the four groups were made using one-way analysis of variance (ANOVA), followed by a Newman–Keuls test. Comparisons of the time course of parameters between the two groups were made using repeated measures two-way ANOVA, followed by a Newman–Keuls test. Survival curves were constructed using the Kaplan–Meier method and compared with log-rank tests. Values of *P*<0.05 were considered statistically significant.

## Results

### Validation of the monocrotaline-induced PAH model

First, we validated the MCT-induced PAH model, the most common experimental model for PAH in rats [24]. As shown in Fig. S1A and S1B, a single i.p. injection of MCT in rats resulted in increases of RV systolic pressure and RV weight, which became significant as early as 2 weeks after MCT injection. These changes were associated with muscularization and wall thickening of pulmonary arterioles (Fig. S1C–D). All PAH rats died between 3–5 weeks after MCT injection, while none of the control rats died during the experimental period. In the present study we used this model to investigate the roles of HMGB1 in MCT-induced PAH.

### Nuclear protein HMGB1 is released to the extracellular space during the early stage of MCT-induced PAH

**Figure 1:**
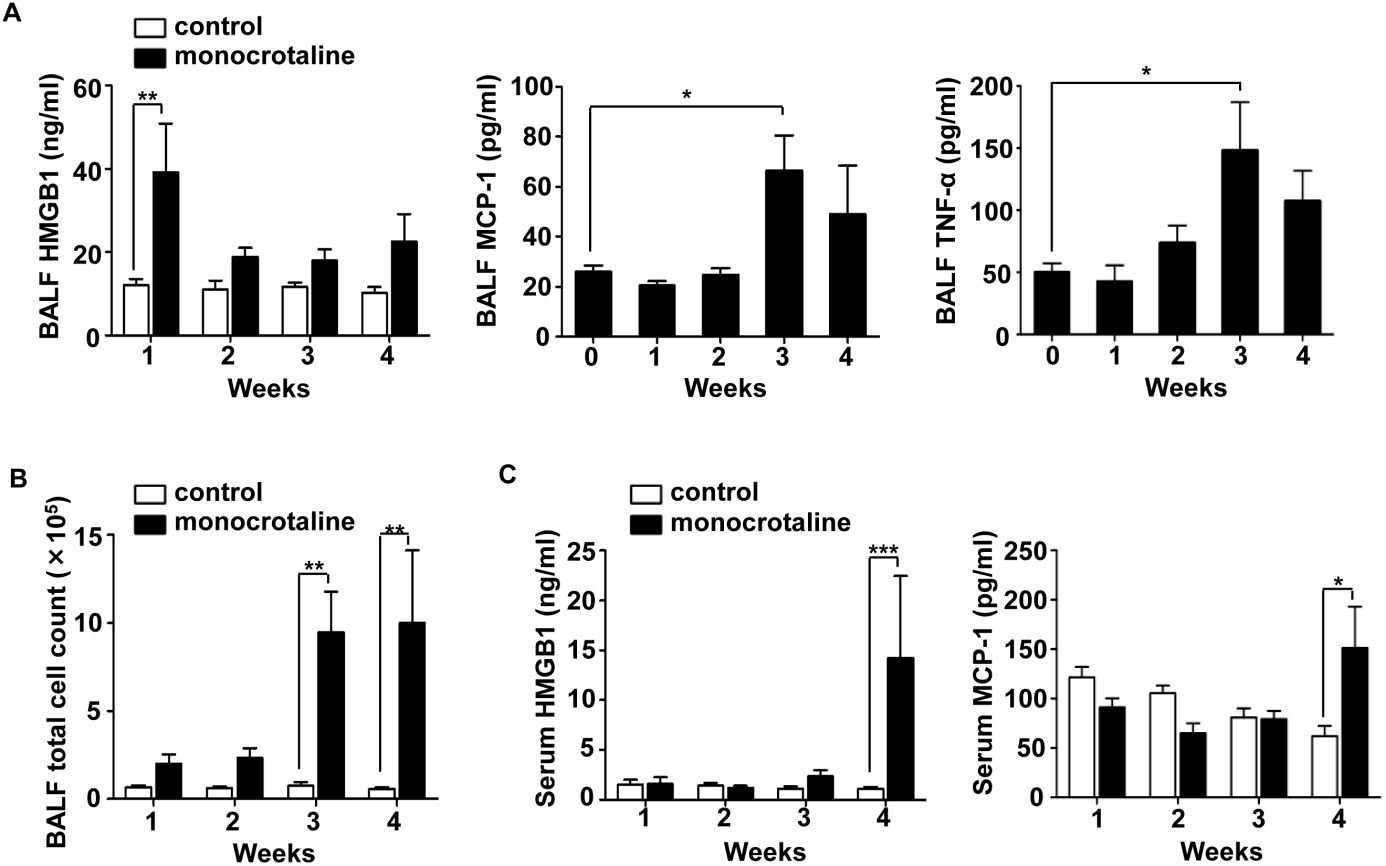
Nuclear protein HMGB1 is released to the bronchoalveolar space during the early stage of PAH. (A–B) BALF samples were collected from MCT- or vehicle-injected rats at the indicated time points. The levels of HMGB1, MCP-1, and TNF-α (A), and the number of leukocytes (B) in BALF were measured. (C) Serum samples were collected from MCT- or vehicle-injected rats, and the levels of HMGB1 and MCP-1 were measured (n = 6–13 per group). All data are expressed as mean ± SEM. *P<0.05, **P<0.01, and ***P<0.001.

Next, we performed time-course analysis of pro-inflammatory cytokine levels, including HMGB1, MCP-1, TNF-α, and IL-1β, in BALF and serum of MCT-induced PAH rats. In response to MCT, nuclear protein HMGB1 was released to the extracellular space, and its concentrations in BALF were significantly increased 1 week after MCT injection (Fig. 1A). MCP-1 and TNF-α levels in BALF were not increased until three weeks after MCT challenge (Fig. 1A). IL-1β levels in BALF were not changed throughout the study (data not shown). Thus, the elevation of HMGB1 in BALF preceded that of other classical pro-inflammatory chemokines and cytokines, such as MCP-1 TNF-α, and IL-1β. The elevation of HMGB1 in BALF also preceded the accumulation of leukocytes in BALF (Fig. 1B), and the development of PAH (Fig. S1). In contrast, serum HMGB1 levels were increased just before PAH rats died (Fig. 1C). Serum MCP-1 levels were also increased at this time point (Fig. 1C), but TNF-α was not detectable in the serum of these rats throughout the study. Thus, HMGB1 is one of the leading pro-inflammatory cytokines in BALF and serum of MCT-induced PAH rats, and may be an attractive target for the treatment of PAH.

**Figure 2:**
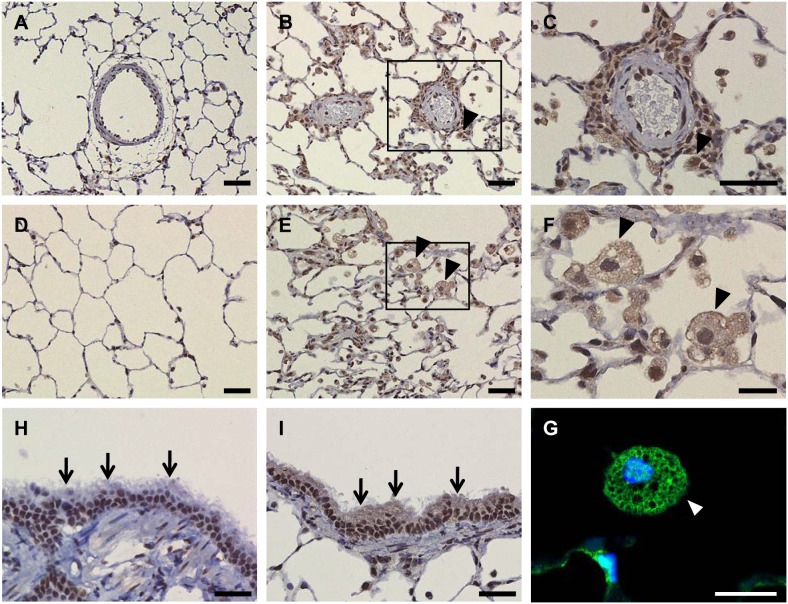
HMGB1 is translocated from the nucleus to the cytoplasm of periarterial infiltrating leukocytes, alveolar macrophages, and bronchial epithelial cells in MCT-injected rats. Lung samples were collected from rats 1 week after vehicle-injection (A, D, H) or MCT-injection (B, C, E–G, I). (C) and (F) are magnified views of the insets shown in (B) and (E), respectively. The localization of HMGB1 was assessed by immunohistochemistry. Nuclear protein HMGB1 was translocated to the cytoplasm of periarterial infiltrating cells, alveolar macrophages (arrowheads), and bronchial epithelial cells (arrows) in MCT-induced PAH rats. Representative images of n = 5–6. Scale bars represent 50 µm (A–E, H, I) and 20 µm (F, G).

To discover the potential cellular sources of HMGB1 in BALF and serum, we examined the localization of HMGB1 in lung tissue. Immunohistochemical analyses showed that HMGB1 was predominantly localized in the nucleus of vascular wall cells (Fig. 2A), alveolar epithelial cells (Fig. 2D), and bronchial epithelial cells (Fig. 2H) in vehicle-injected rats whereas it was translocated to the extranuclear space of periarterial infiltrating cells (Fig. 2B–C), alveolar macrophages (Fig. 2E–G), and bronchial epithelial cells (Fig. 2I) in MCT-injected rats. Thus, periarterial infiltrating cells, alveolar macrophages, and bronchial epithelial cells may be possible cellular sources of BALF HMGB1 in MCT-induced PAH rats.

### Anti-HMGB1 antibody prevents the development of PAH and improves survival of MCT-injected rats

**Figure 3:**
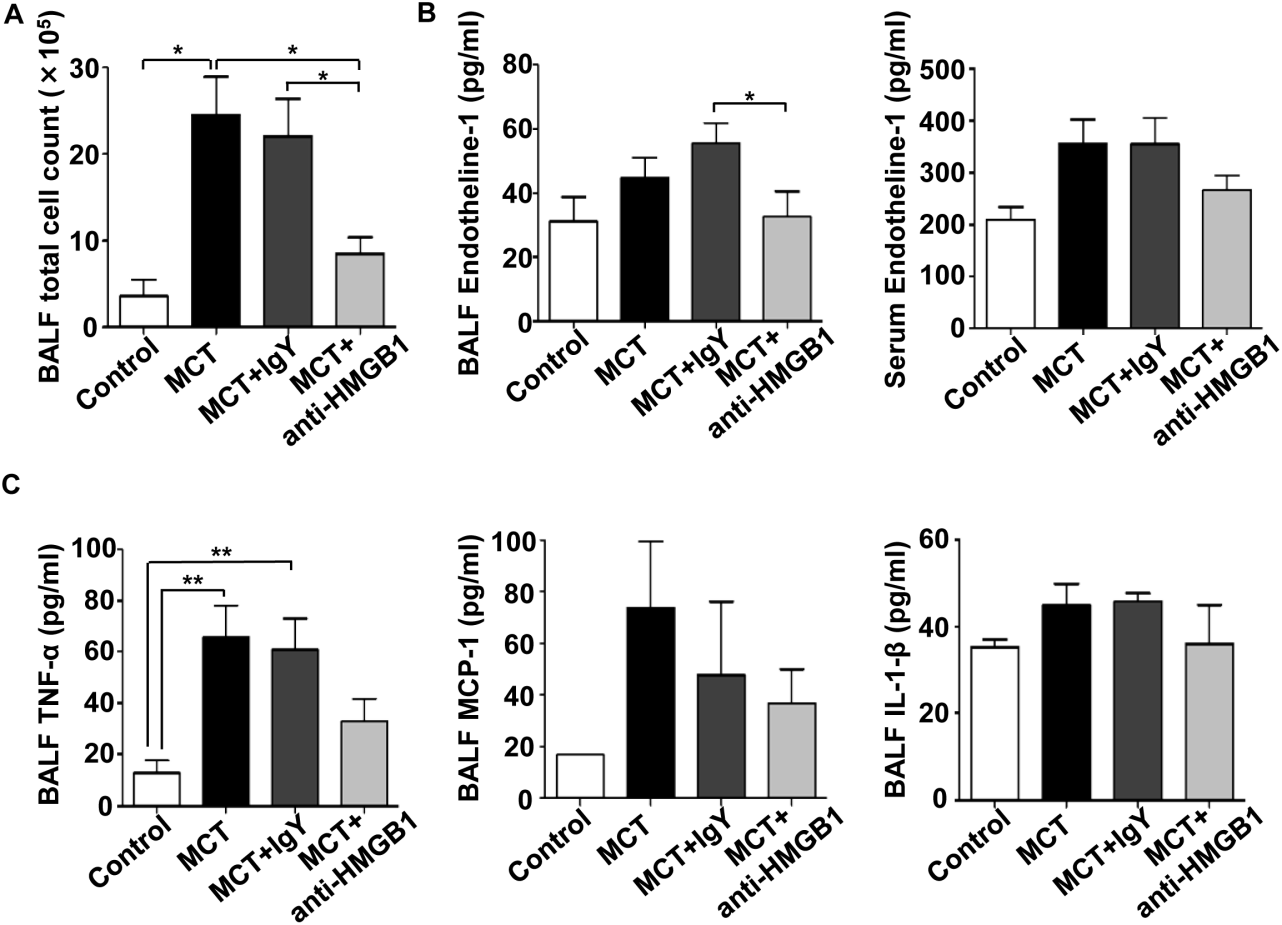
Anti-HMGB1 antibody dampens lung inflammation. BALF samples were collected from MCT-induced PAH rats treated with anti-HMGB1 IgY or control IgY at 3 weeks after MCT challenge. The number of leukocytes (A) and the concentrations of endothelin-1 (B), TNF-α, MCP-1, and IL-1β (C) in BALF were measured (n = 6–7 per group). All data are expressed as mean ± SEM. *P<0.05 and **P<0.01.

**Figure 4:**
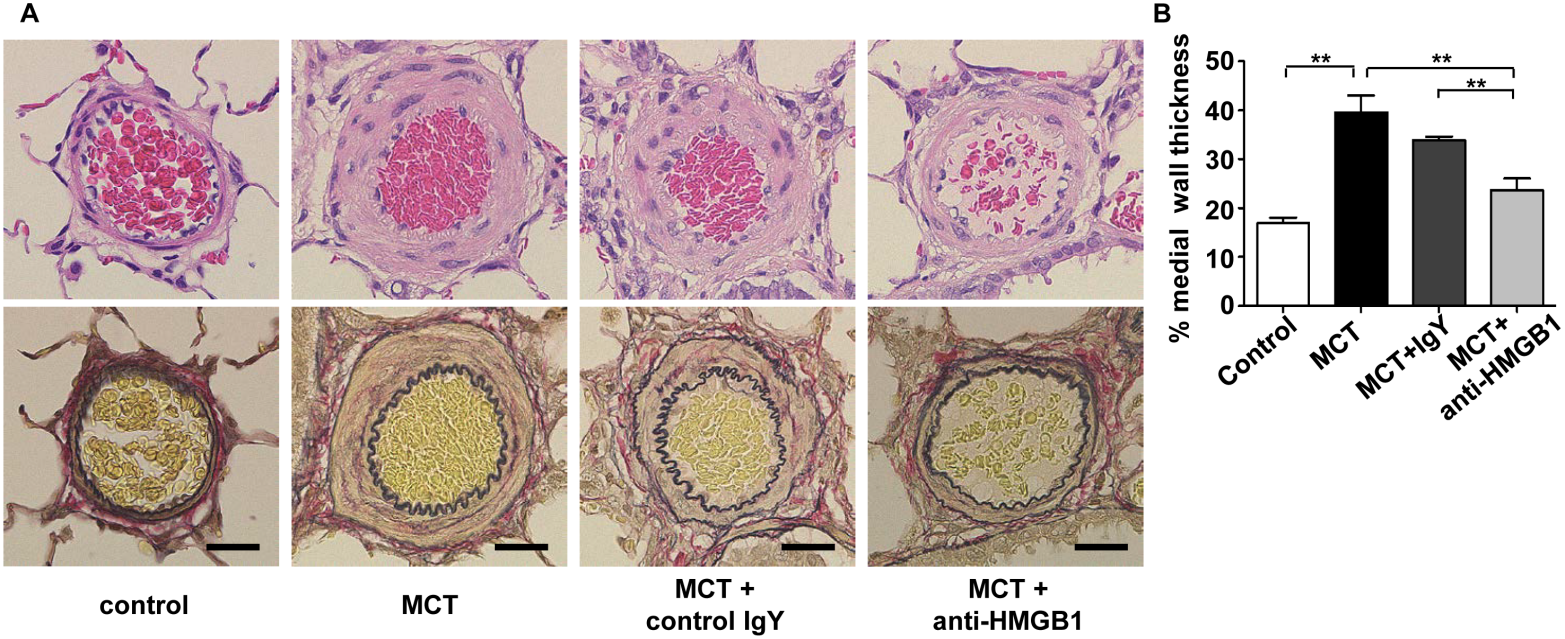
Anti-HMGB1 antibody prevents wall thickening of pulmonary arterioles in MCT-injected rats. (A) H&E staining (upper panels) and Elastica van Gieson staining (lower panels) of pulmonary arteries of MCT-induced PAH rats treated with anti-HMGB1 IgY or control IgY at 3 weeks after MCT challenge. Representative images of n = 6. Scale bars = 20 µm. (B) Pulmonary artery wall thickness were measured in the PAH rats treated with anti-HMGB1 IgY or control IgY at 3 weeks after MCT challenge (n = 6 per group).The external diameter and medial wall thickness of the pulmonary arteries were measured in 20 muscular arteries (ranging in size from 25–100 µm in external diameter) on Elastica van Gieson–stained sections. For each artery, medial wall thickness was expressed as follows: % wall thickness = [(medial thickness×2)/external diameter]×100. All data are expressed as mean ± SEM. **P<0.01.

**Figure 5:**
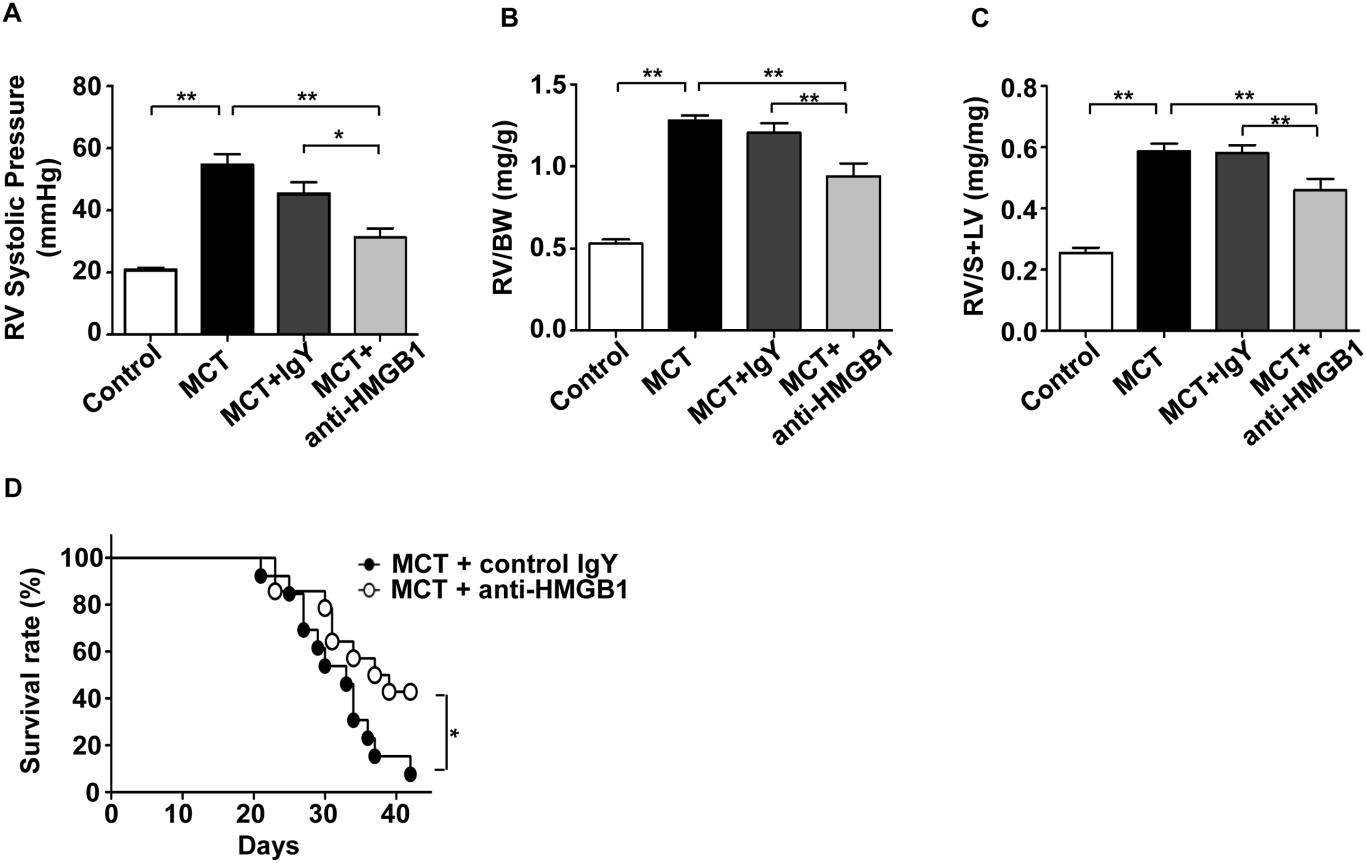
Anti-HMGB1 antibody prevents the development of PAH. RV systolic pressure (A) and RV weight (B–C) of the PAH rats treated with anti-HMGB1 IgY or control IgY at 3 weeks after MCT challenge (n = 6–12 per group). All data are expressed as mean ± SEM. (D) Kaplan-Meier survival curves of the PAH rats treated with either anti-HMGB1 IgY or control IgY (n = 13 per group). *P<0.05 and **P<0.01.

The elevation of pro-inflammatory HMGB1 in the early stage of MCT-induced PAH led us to hypothesize that HMGB1 might be an initiating factor for PAH, and that blocking HMGB1 activity using the anti-HMGB1 antibody could prevent development of PAH. As shown in Fig. 3A, anti-HMGB1 antibody reduced MCT-induced lung inflammation as shown by a decrease in infiltrating leukocytes in BALF. In addition, anti-HMGB1 antibody prevented an increase in BALF endothelin-1 (Fig. 3B), a potent vasoconstrictor with vasoproliferative activity. Anti-HMGB1 antibody tended to decrease BALF TNF-α, MCP-1, and IL-1β levels although the differences did not reach statistical significance (Fig. 3C). Furthermore, anti-HMGB1 antibody prevented muscularization (Fig. S2) and wall thickening of pulmonary arterioles (Fig. 4A–B) in MCT-induced PAH rats. Consequently, anti*-*HMGB1 antibody prevented increases in RV systolic pressure (Fig. 5A) and RV weight (Fig. 5B–C), and improved the survival of MCT-induced PAH rats (Fig. 5D). These findings indicate that extracellular HMGB1 plays an important role in the pathogenesis of MCT-induced PAH, and provide a potential therapeutic target.

## Discussion

Recent studies have suggested that inflammation plays a prominent role in the development of PAH. Pro-inflammatory cytokines and chemokines released by lung resident cells increase vascular permeability and leukocyte infiltration. Infiltrating leukocytes amplify the reactions and release a variety of pro-oxidants and proteolytic enzymes capable of causing lung injury, vasoconstriction, and thrombogenicity; processes implicated in the pathogenesis of pulmonary hypertension [25], [26]. Indeed, expression levels of TNF-α and MCP-1 were increased in the lungs of rats and human patients with PAH, and blocking the activity of these inhibited progression of PAH [26]–[28]. In the current study, we demonstrated that elevation of HMGB1 in BALF preceded that of TNF-α or MCP-1 (Fig. 1A), and blocking the activity of HMGB1 dampened inflammation, prevented the development of PAH, and improved survival in MCT-induced PAH rats (Fig. 3–5). These findings indicate that HMGB1 may act upstream of inflammatory processes in the pathogenesis of PAH, and this damage-associated molecule may govern inflammation and remodeling of the pulmonary vessel wall. Our study has some limitations. A major limitation is that the MCT-induced PAH model does not necessarily reflect the clinical picture of PAH in humans. MCT can damage not only pulmonary arteries but also vasculature of multiple organs, such as liver. In our model, severe liver damage was not evident in histopathological examinations until four weeks after MCT challenge (Fig. S3) although it is still possible that minor damage and inflammatory processes in liver may affect serum cytokine levels and the development of MCT-induced PAH. A recent study indicated that HMGB1 plays a significant role in the development of hypoxia-induced PAH in mice [29], suggesting that the importance of HMGB1 is not limited to a specific model, but might be generally applicable to the pathogenesis of various types of PAH.

HMGB1 can be actively secreted by immunocompetent cells and passively released by damaged cells [30], [31]. In our MCT-induced PAH model, periarterial infiltrating leukocytes, alveolar macrophages, and bronchial epithelial cells might be possible sources of HMGB1 in BALF, because extranuclear HMGB1 was detected in these cells. This idea is consistent with previous reports showing that extranuclear HMGB1 was detected in bronchial epithelial cells and alveolar macrophages after LPS challenge or bleomycin challenge in mice [32], [33]. Another source of extracellular HMGB1 may be endothelial cells [29]. Our *in vitro* study indicated that MCT pyrrole, an active form of MCT *in vivo*, stimulated endothelial cells to release HMGB1 (data not shown). Considering that endothelial injury is a well-established process in the pathophysiology of PAH [8], damaged or activated endothelial cells may be a source of extracellular HMGB1 in MCT-induced PAH rats.

Extracellular HMGB1 acts on RAGE and toll-like receptors expressed on the surface of target cells, such as monocytes and smooth muscle cells [34], [35]. By doing so, HMGB1 promotes inflammation [36], thrombus formation [11], [18], and chemotaxis and proliferation of smooth muscle cells [37], all of which are related to the pathogenesis of PAH. Accordingly, anti-HMGB1 antibody attenuated inflammation (Fig. 3) and muscularization of pulmonary arteries (Fig. S2). Anti-HMGB1 antibody also reduced BALF levels of endothelin-1 (Fig. 3B), a potent vasoconstricting and mitogenic factor for smooth muscle cells and a well-established therapeutic target for PAH. Anti-HMGB1 antibody tended to decrease serum levels of endothelin-1 although the difference did not reach statistical significance. This may be because blood samples were collected from caudal vena cava of PAH rats and the difference might have been more pronounced if blood samples had been collected from the pulmonary vasculature.

HMGB1 is one of the best-characterized damage-associated molecular patterns (DAMPs). DAMPs are released into the extracellular space in response to stress. Once in the extracellular milieu, DAMPs act on surrounding cells, assisting adaption of tissues to stress conditions. Adaptation often provides short-term benefits; however, if stress conditions are sustained or excessive, it can become maladaptive [5], [38]. Specifically, HMGB1 could be released into the extracellular space in response to stress caused by MCT (Fig. 1) or hypoxia [29], and extracellular HMGB1 could promote tissue regeneration [39]. However, sustained or excessive release of HMGB1 might result in remodeling of the pulmonary vessel wall and elevation of pulmonary vascular resistance. Increased HMGB1 levels in serum or plasma reflect systemic inflammation and multiple organ failure and thus are considered to be a predictor of death [40], [41]. In this regard, excessive HMGB1 is detrimental and could be a potential therapeutic target in PAH.

## Supporting Information

Figure S1 **Validation of the MCT-induced PAH model.** (A–B) SD rats were given a single intraperitoneal injection of 60 mg/kg MCT or vehicle, and RV systolic pressure (A) and RV weight (B) were measured 1, 2, 3, or 4 weeks after MCT challenge (n = 6–10 per group). (C–D) H&E staining and Elastica van Gieson staining of lung tissue sections at the indicated time points after MCT injection. The % medial wall thickness was calculated as [(medial thickness×2)/external diameter]×100. Scale bars = 20 µm. All data are expressed as mean ± SEM. **P<0.01 and ***P<0.001.
(TIF)

Figure S2 **Anti-HMGB1 antibody prevents muscularization of pulmonary arterioles in MCT-injected rats.** Elastica van Gieson staining (upper panels) and α-smooth muscle actin immunostaining (lower panels) of pulmonary arteries of MCT-induced PAH rats treated with anti-HMGB1 IgY or control IgY at 3 weeks after MCT challenge. Representative images of n = 6. Scale bars = 20 µm.
(TIF)

Figure S3 **Liver damage in MCT-injected rats.** H&E staining of liver tissue sections at the indicated time points after MCT injection. Representative images of n = 4. Scale bars = 20 µm.
(TIF)
