# Supplementary figures and images for: HMGB1 Promotes the Development of Pulmonary Arterial Hypertension in Rats
Source: PLoS One. 2014 Jul 17;9(7):e102482. doi: 10.1371/journal.pone.0102482 (PMC4102514; doi:10.1371/journal.pone.0102482)

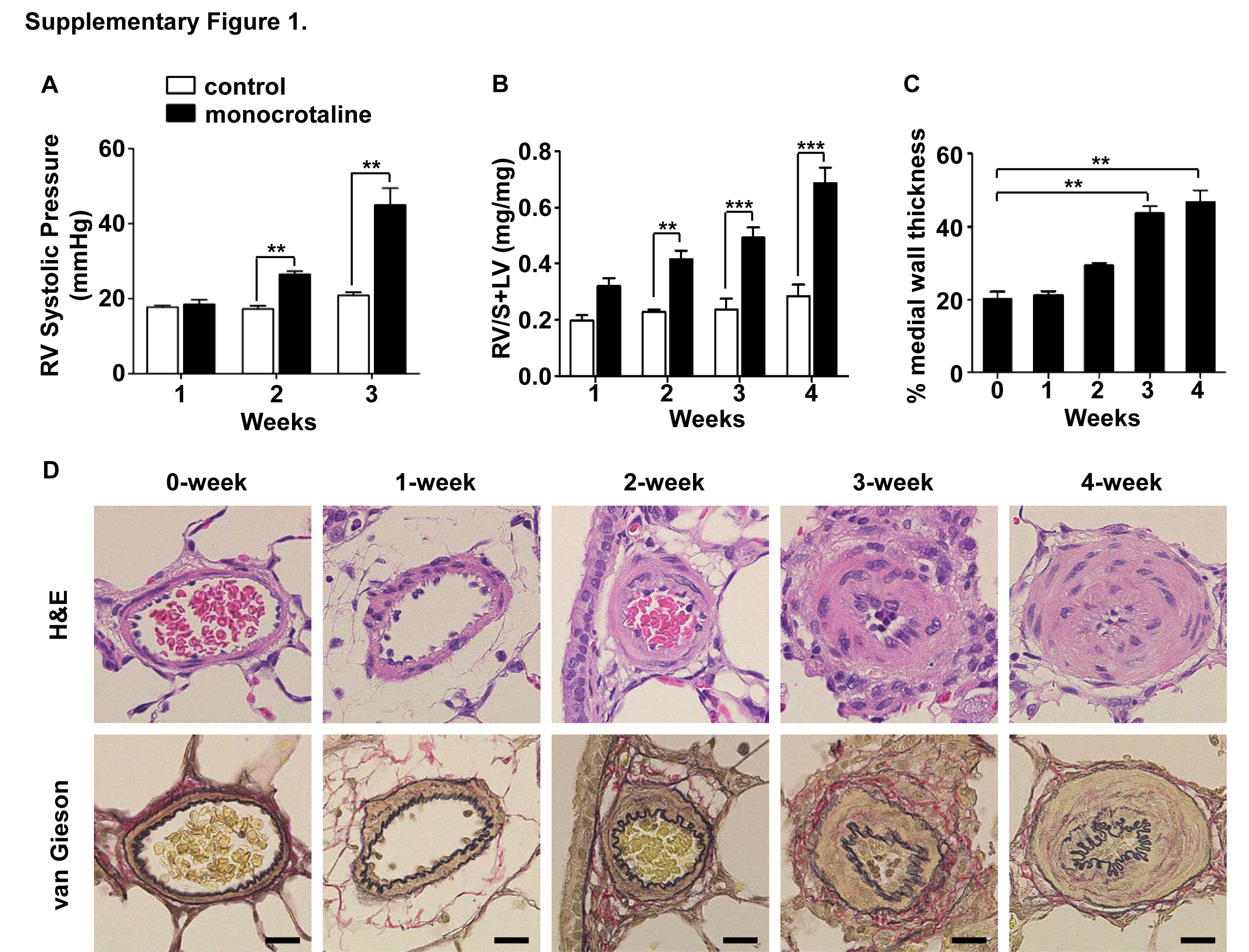

Supplement: Figure S1 — Validation of the MCT-induced PAH model. (A–B) SD rats were given a single intraperitoneal injection of 60 mg/kg MCT or vehicle, and RV systolic pressure (A) and RV weight (B) were measured 1, 2, 3, or 4 weeks after MCT challenge (n = 6–10 per group). (C–D) H&E staining and Elastica van Gieson staining of lung tissue sections at the indicated time points after MCT injection. The % medial wall thickness was calculated as [(medial thickness×2)/external diameter]×100. Scale bars = 20 µm. All data are expressed as mean ± SEM. **P<0.01 and ***P<0.001. (TIF) [file pone.0102482.s001.tif]

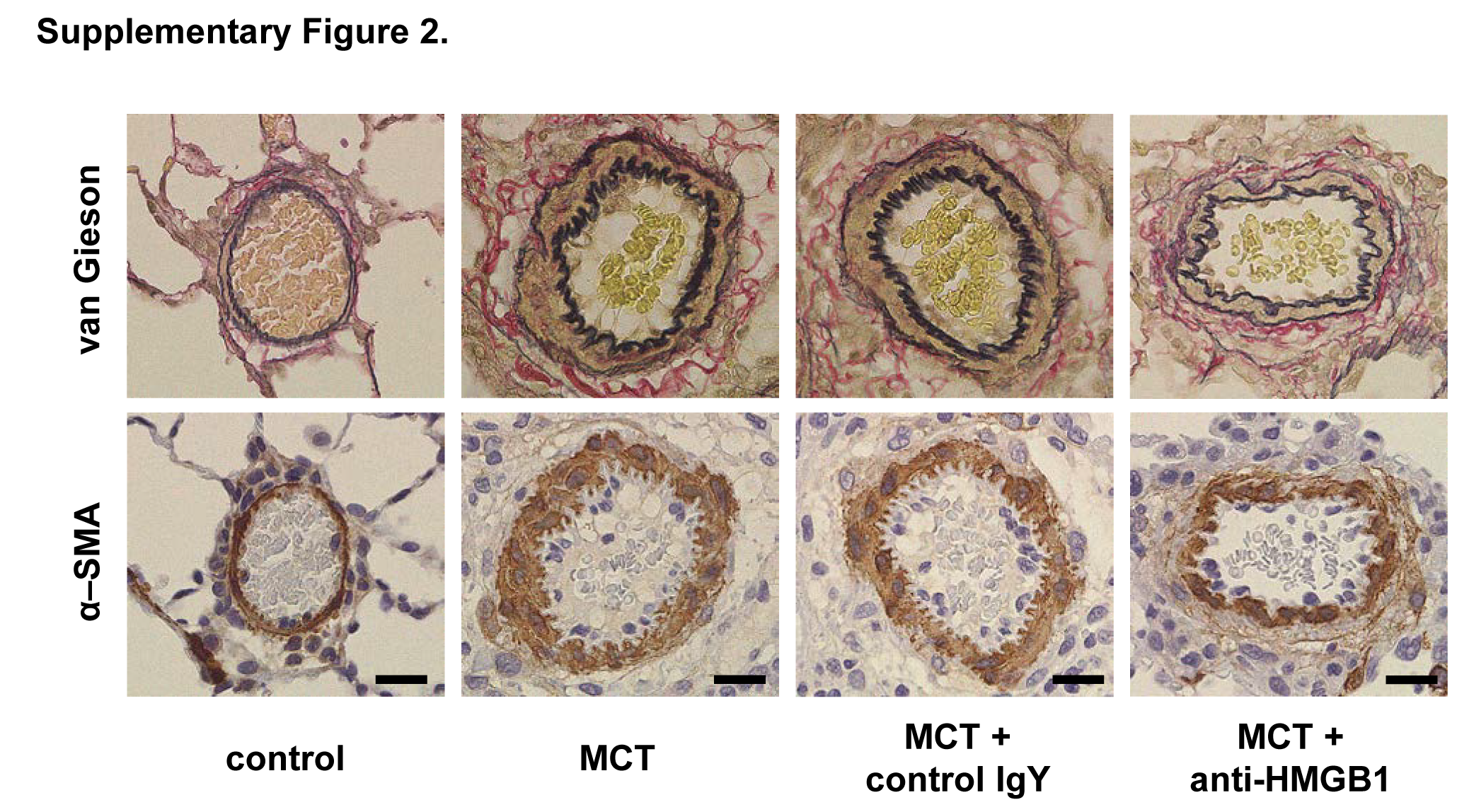

Supplement: Figure S2 — Anti-HMGB1 antibody prevents muscularization of pulmonary arterioles in MCT-injected rats. Elastica van Gieson staining (upper panels) and α-smooth muscle actin immunostaining (lower panels) of pulmonary arteries of MCT-induced PAH rats treated with anti-HMGB1 IgY or control IgY at 3 weeks after MCT challenge. Representative images of n = 6. Scale bars = 20 µm. (TIF) [file pone.0102482.s002.tif]

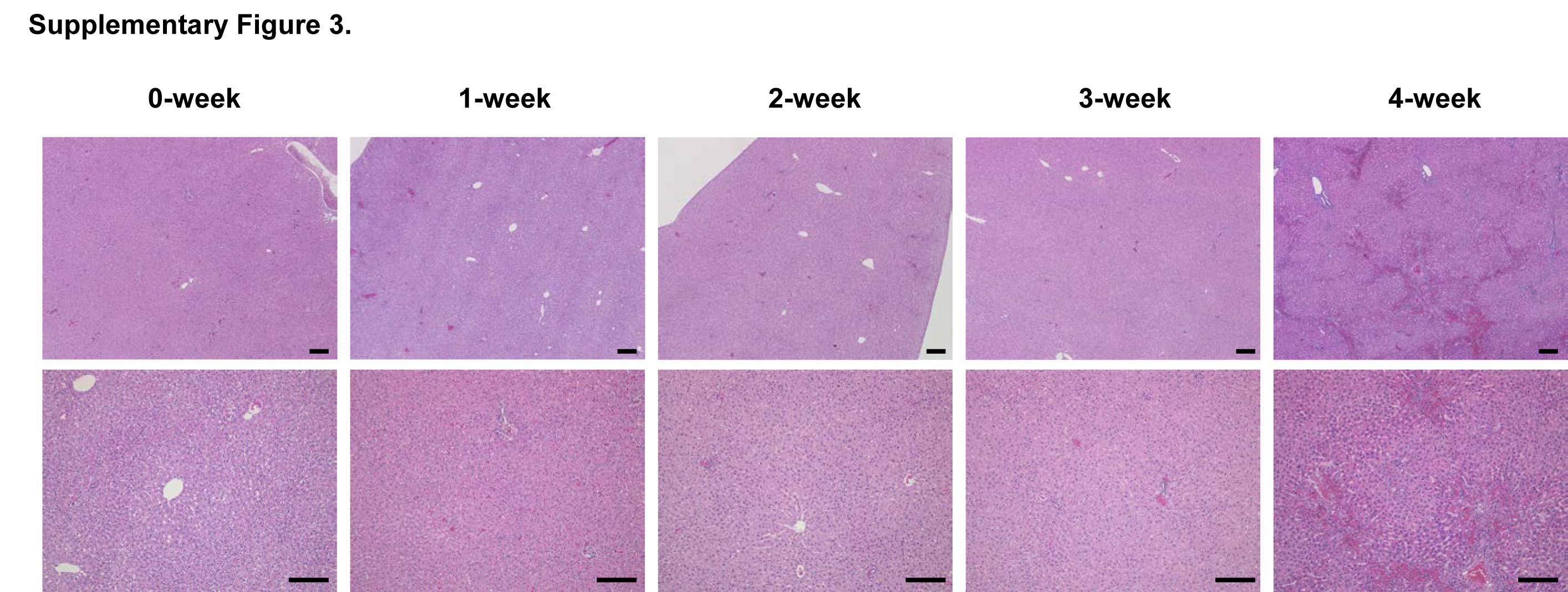

Supplement: Figure S3 — Liver damage in MCT-injected rats. H&E staining of liver tissue sections at the indicated time points after MCT injection. Representative images of n = 4. Scale bars = 20 µm. (TIF) [file pone.0102482.s003.tif]
